# Prognostic factors and the necessity of chemotherapy for stage II gastric cancer: a model based on multicenter retrospective study

**DOI:** 10.1007/s12672-023-00663-w

**Published:** 2023-05-08

**Authors:** Jiaming Fang, Feiyang Zhang, Jun Lu, Zijian Deng, Xianzhe Li, Xijie Chen, Changming Huang, Yingbo Chen, Lei Lian, Junsheng Peng, Shi Chen

**Affiliations:** 1 Department of Gastric Surgery, Department of General Surgery, The Sixth Affiliated Hospital, Sun Yat-sen University, No 26, YuanCun ErHeng Road, TianHe District, Guangzhou, 510655 People’s Republic of China; 2 Guangdong Provincial Key Laboratory of Colorectal and Pelvic Floor Diseases, The Sixth Affiliated Hospital, Sun Yat-sen University, Guangzhou, 510655 China; 3 Department of Gastric Surgery, State Key Laboratory of Oncology in South China, Collaborative Innovation Center for Cancer Medicine, Sun Yat-sen University Cancer Center, Guangzhou, China; 4 Department of Gastric Surgery, Fujian Medical University Union Hospital, No. 29 Xinquan Road, Fuzhou, 350001 China

**Keywords:** Gastric cancer, Adjuvant chemotherapy, Prognosis, Stage II

## Abstract

**Background:**

This study aimed to construct a prognostic model for prognosis prediction and assess the response to adjuvant chemotherapy (ACT) of stage II gastric cancer (GC) patients on high and low survival risk stratifications.

**Methods:**

We retrospectively reviewed 547 stage II gastric cancer patients who underwent D2 radical gastrectomy from January 2009 to May 2017 in Sixth Affiliated Hospital of Sun Yat-Sen University (SAH-SYSU), the Fujian Medical University Union Hospital (FJUUH), and the Sun Yat-Sen University Cancer Center (SYSUCC).The propensity score matching (PSM) of all variables was performed to balance selective bias between ACT and surgery alone (SA) groups. Kaplan–Meier survival and multivariate Cox regression analyses were carried out to identify independent prognostic factors. Independent factors selected by the Cox regression were integrated into the nomogram. The nomogram points stratified patients into high-risk and low-risk groups by the optimal cut-off value.

**Results:**

278 patients were selected after PSM. Age, tumor site, T stage and lymph-nodes-examined (LNE) selected by Cox regression as independent prognostic factors were integrated into the nomogram. The nomogram performed well with a C-index of 0.76 and with C-indexes of 0.73 in and 0.71 in two validate cohorts. AUCs of the 3 year and 5 year ROC curves were 0.81 and 0.78. High- and low-risk groups stratified by the cut-off value demonstrated different responses to ACT.

**Conclusions:**

The nomogram performed well in prognosis prediction. Patients in high- and low-risk groups demonstrated different responses to ACT, and high-risk patients might need ACT.

**Supplementary Information:**

The online version contains supplementary material available at 10.1007/s12672-023-00663-w.

## Introduction

Although a declining trend has been seen in recent decades, gastric cancer (GC) still has the fifth highest incidence and fourth highest mortality of malignant tumors worldwide, particularly in East Asia, Eastern Europe, and South America [1–3]. In Asia, radical gastrectomy of D2 lymph node dissection is the principal surgery for patients with local advanced gastric cancer and has been proven effective in clinical studies of Eastern and Western countries [4–7]. Some randomized controlled trials have proven that radiotherapy does not improve the survival outcomes of GC patients [8, 9]. Chemotherapy is an effective way to improve patients’ prognoses [10–15]. Postoperative adjuvant chemotherapy has been used to eliminate subclinical tumors after surgery, decrease the probabilities of relapse and metastasis and improve the survival rate.

Stage II GC is classified as an earlier advanced stage that typically requires adjuvant chemotherapy. In 2017, the American Joint Committee on Cancer/International Union Against Cancer published the 8th edition of the gastric cancer staging system [16], in which stage II was defined as IIA (T_1_N_2_M_0_, T_2_N_1_M_0_, T_3_N_0_M_0_) and IIB (T_1_N3_a_M_0_, T_2_N_2_M_0_, T_3_N_1_M_0_, T_4a_N_0_M_0_).

Although there have been few trials specifically designed for Stage II GC, subset analyses of randomized controlled clinical trials have shown that adjuvant chemotherapy can be beneficial for these patients [12–15]. The CLASSIC and ACTS-GC studies reported a positive outcome in postoperative adjuvant chemotherapy in stage II GC patients. However, the CLASSIC and ACTS-GC studies did not included patients of T_2_N_0_M_0_ and T_1_N_1_M_0_, which were included in AJCC 8^th^ stage II. However, there is a need for further research to fully analyze the benefit of adjuvant chemotherapy for Stage II GC, particularly those with D2 surgery. According to the National Comprehensive Cancer Network (NCCN) practice guidelines [17] and the Chinese clinical guidelines from the Chinese Society of Clinical Oncology (CSCO) [18], S-1 alone or the XELOX (Xeloda and Oxaliplatin) regimen was first recommended for stage II GC as adjuvant chemotherapy. However, according to previous studies [19–21], a certain number of patients with stage II GC seemed not appear to acquire benefit from ACT, and ACT might even show an adverse effect on prognosis. As such, there is a need to refine the indications for postoperative adjuvant chemotherapy for Stage II GC patients. This multicenter retrospective study aimed to identify independent factors related to overall survival and develop a prognosis-related scoring model to evaluate the prognostic possibility and assess the indication of adjuvant chemotherapy for Stage II GC patients after D2 radical resection. As retrospective studies are prone to missing clinicopathological information and other low-quality data, we utilized the K-nearest neighbor imputation method to interpolate for missing data and performed propensity score matching to mitigate any potential bias arising from subgroup differences.

## Patients and methods

### Patients

In this retrospective study, three independent cohorts, consisted of 547 patients who received curative surgery from January 2009 to May 2017, were enrolled from the databases of the Sixth Affiliated Hospital of Sun Yat-Sen University (SAH-SYSU), the Fujian Medical University Union Hospital (FJUUH), and the Sun Yat-Sen University Cancer Center (SYSUCC). Patients enrolled principally received more than 6 cycles fluoropyrimidine-based postoperative adjuvant chemotherapy regimens, including the S-1 monotherapy (40 mg/m^2^ for S-1, twice daily on Days 1 to 14, with a cycle length of 21 days); Xeloda monotherapy (1000 mg/m^2^,twice daily on Days 1 to 14, with a cycle length of 21 days); XELOX regimen (oxaliplatin at a dose of 130 mg/m^2^ on Day 1, and Xeloda at a dosage of 1000 mg/m^2^ twice daily from Day 1 to Day 14, with a cycle length of 21 days). Similarly, the SOX regimen was administered with oxaliplatin at a dose of 130 mg/m^2^ on Day 1 and S-1 at 40 mg/m^2^, twice daily on Days 1 to 14, with a cycle length of 21 days. Additionally, FOLFOX consisted of oxaliplatin at a dosage of 130 mg/m^2^ administered intravenously on Day 1, calcium folinate at a dosage of 400 mg/m^2^ administered intravenously on Day 1, and 5-fluorouracil at a dosage of 2400 mg/m^2^ administered continuously over 46 h every 14 days. All patients were observed until death or the final follow-up date in December 2020, ensuring that at least 3 years of actual follow-up occurred.

The inclusion criteria were as follows: (i) pathologically diagnosed with stage II according to the AJCC 8th edition TNM system; (ii) histologically confirmed adenocarcinoma of the stomach or esophagogastric junction; and (iii) age over 18 years. (iv) R0 resection with D2 lymph node dissection.

The exclusion criteria were as follows: (i) patients who received preoperative neoadjuvant therapy; (ii) patients with postoperative complications of Clavien–Dindo Classification IV or above; (iii) previous stage II patients not included in AJCC 8th stage; and (iv) patients with incomplete categorical variables and/or missing rate of numeric data > 10%.

The candidate variables were age, gender, carcinoembryonic antigen (CEA), Carbohydrate antigen19-9 (CA19-9), hemoglobin (HB), tumor site, tumor size, tumor differentiation, signet ring cell/mucinous carcinoma (SRCC/MC), perineural invasion status (PNI), lymph-vascular invasion status (LVI), pathological tumor (T) stage (T_1-3_ vs. T_4a_), pathological node (N) stage [negative (N-) vs. positive(N +)], LNE, ACT treatment.

### K nearest neighbor imputation

K nearest neighbor imputation is a commonly used supervised learning method. The imputation principle is to impute some missing data according to a certain number of sample features (number = k) that most resemble the missing value. In this study, either the median (in case of numeric variables) or the most frequent value (in case of categorical variables) of 20 features closest to missing values were chosen for imputation(Shown in supplementary table 1).

### Propensity score matching

To balance ACT and SA groups, propensity score matching of all variables was conducted at the ratio of 1: 1 according to the logistic regression estimated propensity score, and all variables included were analyzed for matching. The R package ‘nonrandom’ was used for PSM.

### Construction, validation, and risk stratification of the nomogram

The nomogram model was constructed using the PSM cohort, and the original cohort with 547 patients was used for validation. Besides, to evaluate the performance of the nomogram model, a cohort of 375 patients from the Surveillance Epidemiology and End Results (SEER) database (SEER Research Plus data, 12 Regs, Nov 2021 sub) [22] was used for external validation, tumor sites in SEER were converted to upper (C16.0-Cardia, NOS and C16.1-Fundus of stomach), middle (C16.2-Body of stomach), lower (C16.3-Gastric antrum and C16.4-Pylorus) and overlap (C16.8-Overlapping lesion of stomach). C-index, calibration curves, area under receiver operating characteristic curves and DCA curves were used to evaluate the performance of the nomogram. The risk score of each patient was obtained from the nomogram. The optimal cut-off value of the nomogram total points was calculated by the maximal Youden Index from the 5 year ROC curve, and patients were stratified into the high-risk group and the low-risk group by the optimal cut-off value.

### Statistical analysis

Continuous variables are demonstrated as median ± 25th and 75th percentiles, and categorical variables as percentages. Statistic differences in variables between two groups were analyzed by using chi-squared tests for categorical variables and parametric tests for numeric data subjecting to the normal distribution or nonparametric tests for numeric data not subjecting to normal distribution.

The survival analysis was performed by using the Kaplan–Meier method and log-rank test if indicated. To analyze significantly independent prognostic factors, univariate Cox regression analysis was performed after PSM, followed by the multivariate Cox analysis to evaluate prognostic impact for the factors selected by the univariate Cox analysis. The *P*-value and the hazard ratios (HR) were used to demonstrate the outcome of Cox regression.

For all outcomes of statistical analysis, *P*-value < 0.05 in a two-tailed test was considered statistically. All the statistical analysis processing and picture plotting were conducted using R software (Version 4.1.2). K-nn imputation procedure was performed by R package “DMwR2”. PSM was performed by R package “nonrandom”. The nomogram model was constructed by R packages “survival” and “rms”. Cox regression was performed by R package “survival”.

## Results

### Patient characteristics

**Fig. 1:**
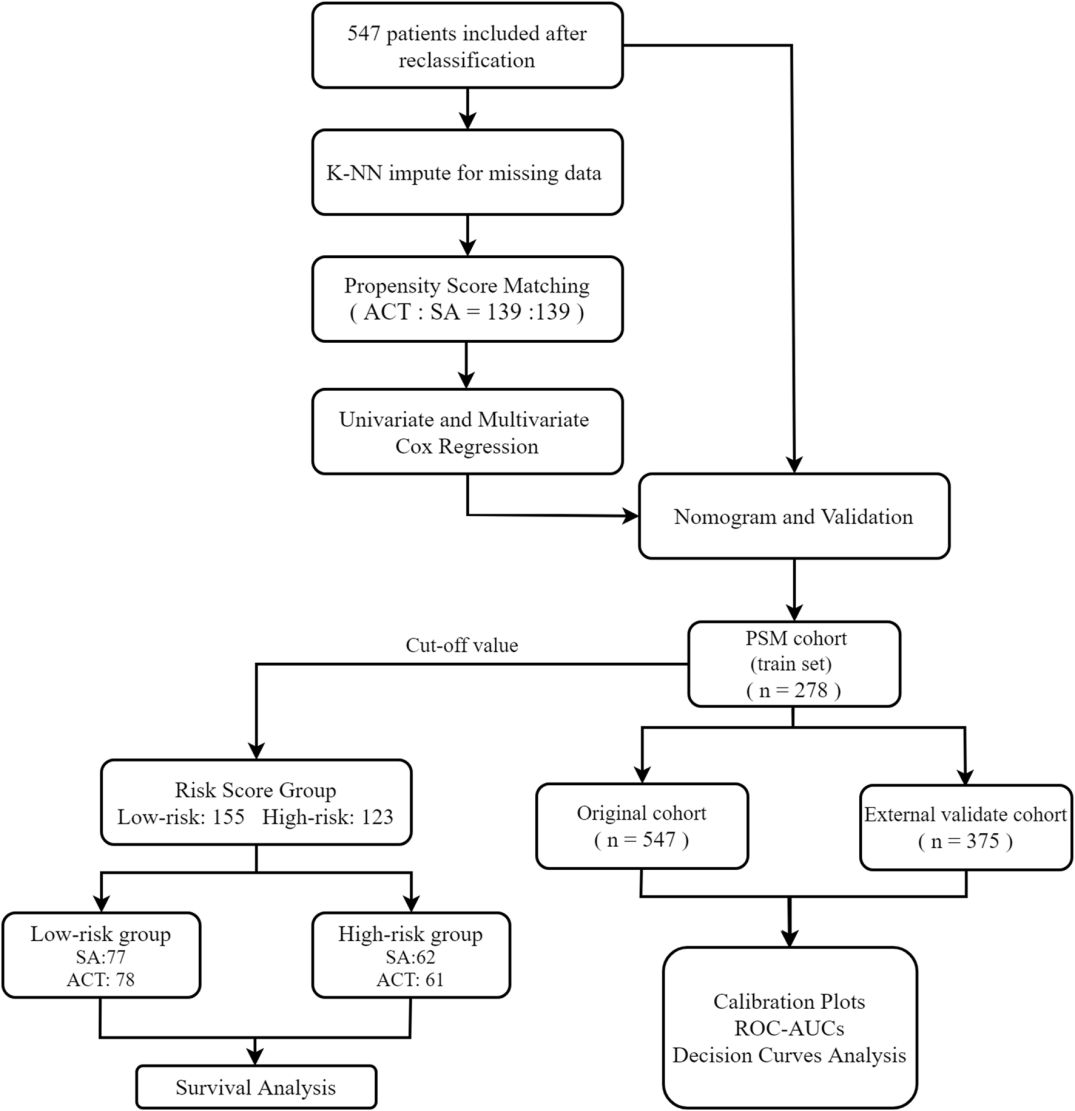
Flowchart of the analysis process

In this study, a total of 547 patients who underwent D2 radical surgery were included (270 patients from the FJUUH, 166 patients from the SYSUCC and 111 patients from the SAH-SYSU). Before PSM, there were far more patients in the ACT group (n = 408) than in the SA group (n = 139). After PSM, all patients in SA group were matched at the ratio of 1: 1. Before performing the propensity score matching (PSM) analysis, a marked discrepancy was observed between the surgery combined with adjuvant chemotherapy group and the surgery alone group in terms of the number of lymph node dissections, particularly concerning age-related differences. Subsequently, following the PSM procedure, we obtained a more balanced distribution of patient demographics between the two groups, thereby mitigating any potential biases. The demographic data and clinicopathological parameters of both groups before and after PSM are summarized in Table 1, and the whole analysis process is demonstrated in Fig. 1. The cut-off values for CEA and CA19-9 were set at 5 ng/ml and 37.0 U/ml, respectively.

**Table 1:** Characteristics of patients with stage II gastric cancer before and after propensity score matching

Characteristics	Before propensity score matching		*P*-value	After propensity score matching		*P*-value
	Surgery alone (n = 139)	Adjuvant chemotherapy (n = 408)		Surgery alone (n = 139)	Adjuvant chemotherapy (n = 139)	
Age (years)	57 (64–70)	51 (60–67)	0.002	64 (57–70)	62 (57–69.50)	0.89
Sex			0.30			0.88
Male	108 (77.7)	297 (72.8)		108 (77.7)	110 (79.1)	
Female	31 (22.3)	111 (27.2)		31 (22.3)	29 (20.9)	
CEA (ng/ml)	2.24 (1.44–3.90)	1.37 (2.20–3.40)	0.44	2.24 (1.44–3.90)	2.50 (1.75–3.82)	0.19
CA19-9 (U/ml)	9.15 (4.64–17.90)	9.02 (4.71–21.60)	0.72	9.15 (4.64–17.90)	9.26 (5.82–21.70)	0.42
Hemoglobin (g/L)	129 (106–141)	126 (108–139)	0.38	129 (106–141)	129 (111–144)	0.68
Tumor stie			0.60			0.97
Upper	47 (33.8)	114 (27.9)		47 (33.8)	44 (31.6)	
Middle	18 (12.9)	62 (15.2)		18 (12.9)	20 (14.4)	
Lower	67 (48.2)	208 (51.0)		67 (48.2)	67 (48.2)	
Overlap	7 (5.1)	24 (5.9)		7 (5.1)	8 (5.8)	
Tumor Size (cm)	4.00 (3.00–5.50)	4.00 (3.00–5.00)	0.73	4.00 (3.00–5.50)	4.00 (3.00–5.00)	0.78
Differentiation			0.55			0.81
Well/moderately differentiated	78 (56.1)	215 (52.7)		78 (56.1)	81 (58.3)	
Poorly differentiated	61 (43.9)	193 (47.3)		61 (43.9)	58 (41.7)	
Signet cell/Mucinous carcinoma			0.87			0.51
No	115 (82.7)	333 (81.6)		115 (82.7)	120 (86.3)	
Yes	24 (17.3)	75 (18.4)		24 (17.3)	19 (13.7)	
Perineural invasion			0.80			1.00
No	90 (64.7)	271 (66.4)		90 (64.7)	89 (64.0)	
Yes	49 (35.3)	137 (33.6)		49 (35.3)	50 (36.0)	
Lymph-vascular invasion			0.36			0.85
No	122 (87.8)	343 (84.1)		122 (87.8)	124 (89.2)	
Yes	17 (12.2)	65 (15.9)		17 (12.2)	15 (10.8)	
T stage			0.26			0.87
T1-T3	118 (84.9)	363 (89.0)		118 (84.9)	116 (83.5)	
T4a	21 (15.1)	45 (11.0)		21 (15.1)	23 (16.5)	
N stage			0.63			0.81
N-	70 (50.4)	217 (53.2)		70 (50.4)	73 (52.5)	
N +	69 (49.6)	191 (46.8)		69 (49.6)	66 (47.5)	
Lymph nodes examined	36 (26.50–45)	29 (21–39)	< 0.001	36 (26.50–45)	34 (25–44.5)	0.65

### Propensity score matching

Logistic regression was utilized to determine the propensity score of each patient, and based on the closest propensity score, patients were matched. Patients in the ACT group exhibited significant differences in age (p = 0.002) and LNE (p < 0.001) compared to the SA group, with a larger sample size. Subsequent 1:1 all-variable propensity score matching was applied to reduce major bias (Caliper = 0.05), resulting in the eventual balance of all covariates between the two groups (Table 1 and Supplementary Figure 1). Ultimately, PSM selected 278 patients.

### Univariate and multivariate Cox regression

**Fig. 2:**
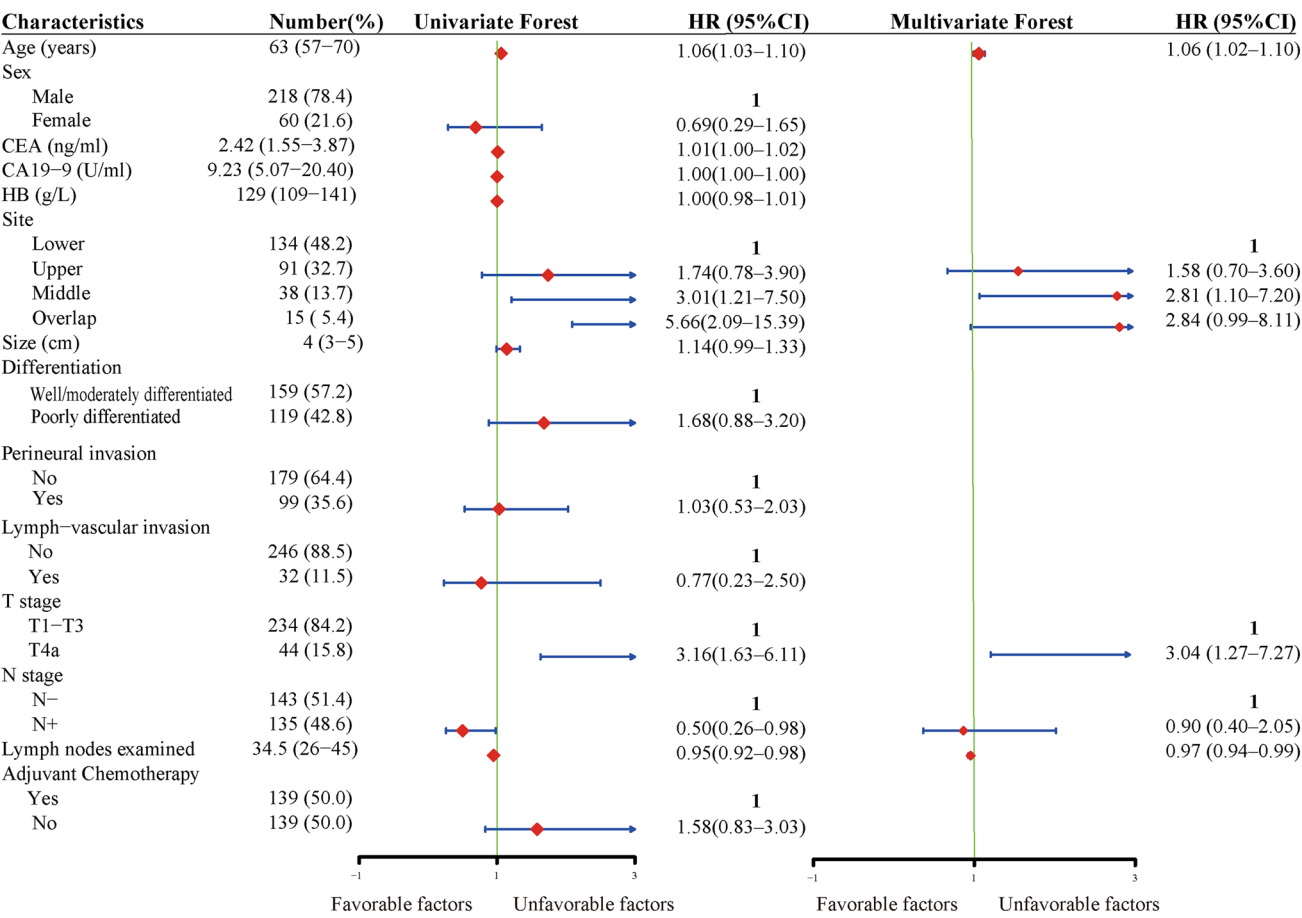
Forest plots of univariate and multivariate cox regression of the PSM cohort

Univariate Cox regression of the PSM cohort revealed that age (p < 0.001), tumor site (p < 0.001), pathological T stage (p < 0.001), pathologic N stage (p = 0.04), and LNE (p < 0.001) were identified as prognosis-related factors. Age (p = 0.001), tumor site (p = 0.03), pathological T stage (p = 0.01) and LNE (p = 0.01) were subsequently identified as independent factors via multivariate Cox regression (Table 2 and Fig. 2).

**Table 2:** Univariate and multivariate Cox regression

Characteristics	Number (%)	Univariate Cox regression		Multivariate Cox regression	
		HR (95% CI)	*P*-value	HR (95% CI)	*P*-value
Age (years)	63 (57–70)	1.06 (1.03–1.10)	< 0.001	1.06 (1.02–1.10)	0.001
Sex			0.40		
Male	218 (78.4)	1			
Female	60 (21.6)	0.69 (0.29–1.65)			
CEA (ng/ml)	2.42 (1.55–3.87)	1.01 (1.00–1.02)	0.07		
CA19-9 (U/ml)	9.23 (5.07–20.40)	1.00 (1.00–1.00)	0.78		
Hemoglobin (g/L)	129 (109–141)	1.00 (0.98–1.01)	0.68		
Tumor stie					
Upper	91 (32.7)	1.74 (0.78–3.90)	0.18	1.58 (0.70–3.60)	0.27
Middle	38 (13.7)	3.01 (1.21–7.50)	0.02	2.81 (1.10–7.20)	0.03
Lower	134 (48.2)	1		1	
Overlap	15 (5.4)	5.66 (2.09–15.39)	< 0.001	2.84 (0.99–8.11)	0.05
Tumor size (cm)	4 (3–5)	1.14 (0.99–1.33)	0.07		
Differentiation			0.12		
well/moderately differentiated	159 (57.2)	1			
poorly differentiated	119 (42.8)	1.68 (0.88–3.20)			
Signet cell/Mucinous carcinoma			0.04		0.13
No	235 (84.5)	1		1	
Yes	43 (15.5)	2.13 (1.03–4.41)		1.77 (0.84–3.72)	
Perineural invasion			0.92		
No	179 (64.4)	1			
Yes	99 (35.6)	1.03 (0.53–2.03)			
Lymph-vascular invasion			0.66		
No	246 (88.5)	1			
Yes	32 (11.5)	0.77 (0.23–2.50)			
T stage			< 0.001		0.01
T1-3	234 (84.2)	1		1	
T4a	44 (15.8)	3.16 (1.63–6.11)		3.04 (1.27–7.27)	
N stage			0.04		0.81
N-	143 (51.4)	1		1	
N +	135 (48.6)	0.50 (0.26–0.98)		0.90 (0.40–2.05)	
Lymph nodes examined	34.5 (26–45)	0.95 (0.92–0.98)	< 0.001	0.97 (0.94–0.99)	0.01
Adjuvant chemotherapy			0.17		
Yes	139 (50.0)	1			
No	139 (50.0)	1.58 (0.83–3.03)

### Establishment and validation of the Nomogram

**Fig. 3:**
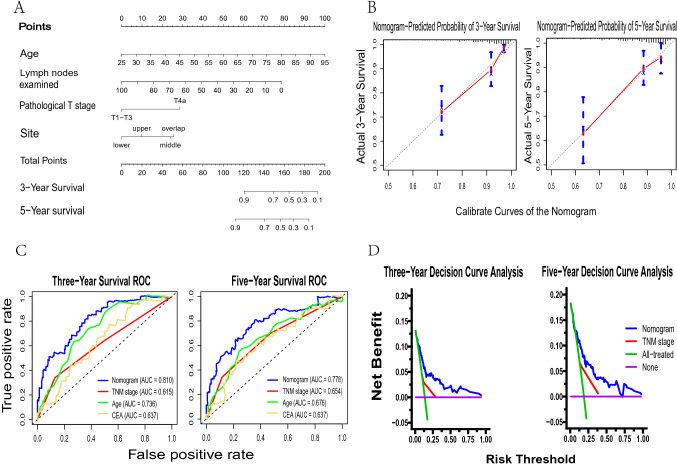
The nomogram for stage II gastric cancer patients after D2 surgery. **A** Nomogram, **B** 3- and 5 year calibration curves of the nomogram and two mixed validate cohorts, **C** 3- and 5 year receiver operating curves of the nomogram and other variables, **D** 3- and 5 year decision curves of the nomogram and the TNM staging system

The nomogram integrated age, tumor site, pathological T stage, and LNE to predict 3 year and 5 year overall survival probabilities of patients after D2 surgery (Fig. 3A). The model’s performance was evaluated using the original cohort of 547 patients and the SEER cohort of 375 patients, with concordance indices of 0.76, 0.73, and 0.71, respectively. Calibration curves demonstrated close agreement between the nomogram-predicted and actual survival rates at 3 and 5 years (Fig. 3B), while receiver operating characteristic curves revealed superior performance of the nomogram in predicting survival over traditional TNM stage and other variables (Fig. 3C). Additionally, the nomogram’s performance was consistent in the SEER and original cohorts (Supplementary Figure 2), as confirmed by 3- and 5 year decision curve analysis curves (Fig. 3D).

### Risk stratification and survival analysis

**Fig. 4:**
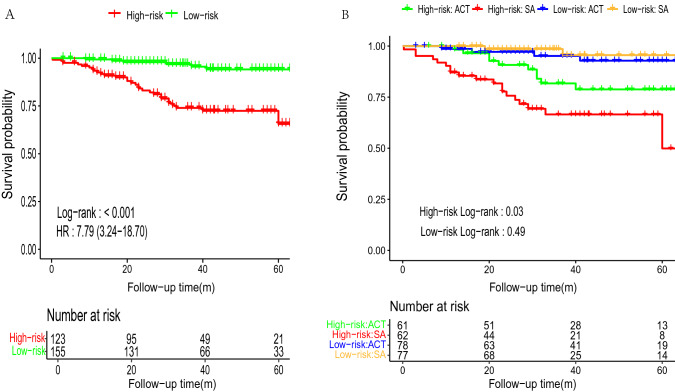
Nomogram stratified Kaplan–Meier curves of the high- and the low-risk groups and subgroups

Nomogram total points were calculated for each patient, ranging from 0.00 to 200.00, with an optimal cut-off value of 117.93 according to the maximum Youden Index of the 5 year ROC curve (Supplementary Figure 3). The high-risk group (123 patients) had higher age, CEA, tumor site overlap, tumor size, T stage, and lower N stage and lymph nodes examined than the low-risk group (155 patients) (Supplementary Table 2). Kaplan–Meier survival curves and Cox regression analysis demonstrated that the high-risk group had a worse prognosis (p < 0.001, HR: 7.79, 95% CI 3.24–18.70) (Fig. 4). Patients in the high-risk group who received ACT had a better prognosis than those who received SA, and ACT was an independent factor for prognosis (p = 0.02, HR (SA vs. ACT) = 2.44, 95% CI 1.13–5.27) (Supplementary Figures 4 and 5). However, there was no significant survival difference between patients in ACT and SA groups in the low-risk group, and ACT was not a significant independent factor (p = 0.43, HR (SA vs. ACT) = 0.50, 95% CI 0.09–2.80), with only one patient with overlap site in this group.

## Discussion

For overall patients with stage II gastric cancer, the NCCN and CSCO guidelines both recommend fluoropyrimidine-based regimens (such as S-1 alone or XELOX) as adjuvant chemotherapy [17, 18]. Certain prospective randomized controlled trials demonstrated the effectiveness of ACT for overall stage II GC patients [12–15]. Indeed, from overall situation, stage II GC patients receiving ACT showed an improved survival rate, but the large-scale randomized controlled trials such as CLACSSIC and ACTS-GC enrolled patients according to the AJCC 6th and 2nd Japanese Gastric Cancer Association staging system. Compare with old staging systems, the latest published AJCC staging system of 8th edition redefined subserosa-invasive AJCC 6th T_2_ stage as AJCC 8th T_3_ stage, and divided N_3_ stage in to N_3a_ stage (7–15 regional positive lymph nodes) and N_3b_ stage (≥ 16 regional positive lymph nodes). Therefore, in the context of the AJCC 8th staging system, previous studies might not provide sufficient evidence to support clinical decisions. A fiercely disputable topic was the patients at the stage of T_3_N_0_M_0_ or T_1_N_2_M_0_. The Japanese Gastric Cancer Association pointed out that patients at the stage of T_1_N_0-3_M_0_ or T_3_N_0_M_0_ were not suggested to undergo adjuvant chemotherapy due to changes of the staging system [23]. It is worth noting that patients with a T3N0 stage according to the eighth edition of the staging system correspond to stage Ib (T2N0) in the sixth edition. The ACTS and CLASSIC studies have been conducted on this particular patient group. However, whether these patients require adjuvant chemotherapy after undergoing radical surgery for D2 gastric cancer remains uncertain. As pT1N0-3 is typically limited to early gastric cancer and is not typically associated with a need for chemotherapy, it has not been included in clinical studies that examine the use of adjuvant chemotherapy after radical gastric cancer surgery. Therefore, at present, neither of these two groups is considered to require postoperative adjuvant chemotherapy in accordance with the Japanese gastric cancer treatment guidelines. Some retrospective studies also demonstrated that a certain number of stage II GC patients might not benefit from ACT. In our previous study, no benefit produced by postoperative adjuvant chemotherapy was shown in patients at the stage of T_3_N_0_M_0_ [19]. Another single center study by Lee KG et al. [20] figured out that adjuvant chemotherapy did not improve the outcome of patients of T_3_N_0_M_0_ or T_1_N_2_M_0_ stage, but a multicenter study conducted by Huang ZN et al. [21] confirmed that some patients of T_3_N_0_M_0_ and T_1_N_2_M_0_ stages might receive benefit from adjuvant chemotherapy. Nevertheless, few studies focus on the overall II stage. With the publishment of AJCC 8th staging system, the range of the pathological stage II GC population was more detailed. Indications for postoperative adjuvant chemotherapy for gastric cancer patients with pathological stage II disease should be further improved.

To predict prognosis is very helpful for the clinical decisions and survival risk stratification. In our study, age, tumor site, pathological T stage and lymph node examined were eventually confirmed as independent prognostic factors by means of univariate and multivariate Cox regression. The nomogram of independent factors exhibited a favorable discrimination and calibration in the PSM cohort, The nomogram for had a satisfactory accuracy in predicting overall survival for stage II GC patients. Two mixed validation cohorts confirmed the model generalizability. A survival risk stratification based on the nomogram was utilized to assess the effectiveness of ACT. Significant survival disparities were shown in the high- and low-risk groups. Meanwhile, significant survival between patients with ACT and SA was shown in the high-risk group but not the low-risk group. Although balancing all variable difference by PSM, ACT showed no significance in univariate Cox regression. While in the high-risk group, univariate and multivariate Cox regression proved that ACT was an independent factor, which indicated that the survival risk strata had good discrimination ability in terms of indication for ACT. And patients with high survival risk might meet the indications of adjuvant chemotherapy.

However, in our model, age is a disputable issue. Elderly patients were not recommended for adjuvant treatment [24–26]. Certain meta-analyses showed that ACT did not benefit elderly patients [27, 28]. Therefore, age and other risk factors in combination with the risk score should be comprehensively considered before adjuvant treatment. Patients with overlap tumor site, especially the linitis plastica were often considered with the worst prognosis, which was in line with the nomogram. The number of lymph nodes examined was also an independent factor in the prognosis of stage II GC, which was consistent with previous studies which indicated that the number of LNE was a prognostic factor for gastric cancer at all stages [29–32]. The more lymph nodes examined, the lower the probability of omitted positive lymph nodes, the more accurate for tumor staging, and eventually the better for cancer treatment. Interestingly, pathological N stage and T stage presented opposite trends in Cox regression. Such an outcome might be attributed to the limitation of pathological staging in the stage II. After PSM to balance bias, pathological T stage was eventually selected as an independent factor by means of multivariate Cox regression. Thus, patients of AJCC 8th stage II with older age, higher T stage and less examined lymph nodes were more likely to obtain benefit from postoperative chemotherapy. However, while our study suggests that older patients may have a better prognosis, it is essential to consider the potential toxic effects of chemotherapy in elderly patients. The decision to administer chemotherapy in elderly patients remains controversial, and caution should be exercised when considering this treatment option.

There are several limitations in our study. First, this is a retrospective study and there remains the probability of data access error and selective bias. Second, disease-free survival (DFS) could serve as a more robust measure for assessing the efficacy of postoperative adjuvant chemotherapy. Nonetheless, we must acknowledge that this study has a retrospective design, and consequently, there were some patients who were not reviewed promptly or did not revisit our hospital for follow-up. These factors impeded our ability to obtain accurate DFS data for each patient. Third, due to the limited number of patients, we grouped patients based on the degree of tumor differentiation, categorizing them as high/moderately differentiated or low differentiated. Lauren typing for gastric cancer was also not included in this study as it was not performed by some of the participating centers. Moreover, the distinction between microvascular infiltration and lymphovascular infiltration was not made by most centers and was generally described as pulsatile infiltration. More studies are needed to reveal the impact of these factors on AJCC 8th stage II GC patient’s prognosis and to select appropriate ACT candidates, and our predictive model needs further improvement.

## Conclusion

In our study, age, tumor site, T stage and LNE selected from univariate and multivariate Cox regression analyses were affirmed to be independent prognostic factors for patients with stage II GC and these four independent factors were used to construct a clinical predictive model. The model performed well in predicting 3 year and 5 year survival rates. In addition, a risk stratification was performed according to the optimal cut-off value of the nomogram total points. The difference of prognosis between ACT and SA subgroups was shown between high- and low-risk group, which indicated that high-risk patients defined by independent factors might benefit from adjuvant chemotherapy.

## Supplementary Information

Supplementary file 1—Figure 1: Numerical variables and categorical variables before and after PSM.

Supplementary file 2—Figure 2: ROC curves of train and validate datasets.

Supplementary file 3—Figure 3: Optimal cut-off value for risk stratification.

Supplementary file 4—Figure 4: Multivariate cox regression forest plot of the high-risk group.

Supplementary file 5—Figure 5: Multivariate cox regression forest plot of the low-risk group.

Supplementary file 6—Table 1: Characteristics before and after imputation. Table 2: Characteristics between high-risk and low-risk groups

## Data Availability

The datasets used and/or analyzed during the current study are available from the corresponding author on reasonable request.

## Abbreviations

AJCC: American Joint Committee on Cancer
ACT: Adjuvant chemotherapy
GC: Gastric cancer
k-NN: K-nearest neighbor
PSM: Propensity score matching
SA: Surgery alone
ROC: Receiver operating characteristic curve
AUC: Area under the curve
LNE: Lymph nodes examined
NCCN: The National Comprehensive Cancer Network
CSCO: The Chinese Society of Clinical Oncology
CEA: Carcinoembryonic antigen
CA19-9: Carbohydrate antigen19-9
HB: Hemoglobin
SRCC/MC: Signet ring cell/mucinous carcinoma
HR: Hazard ratio
