# Supplementary figures and images for: Prognostic factors and the necessity of chemotherapy for stage II gastric cancer: a model based on multicenter retrospective study
Source: Discov Oncol. 2023 May 8;14:58. doi: 10.1007/s12672-023-00663-w (PMC10167061; doi:10.1007/s12672-023-00663-w)

# ACT

■ No ■ Yes

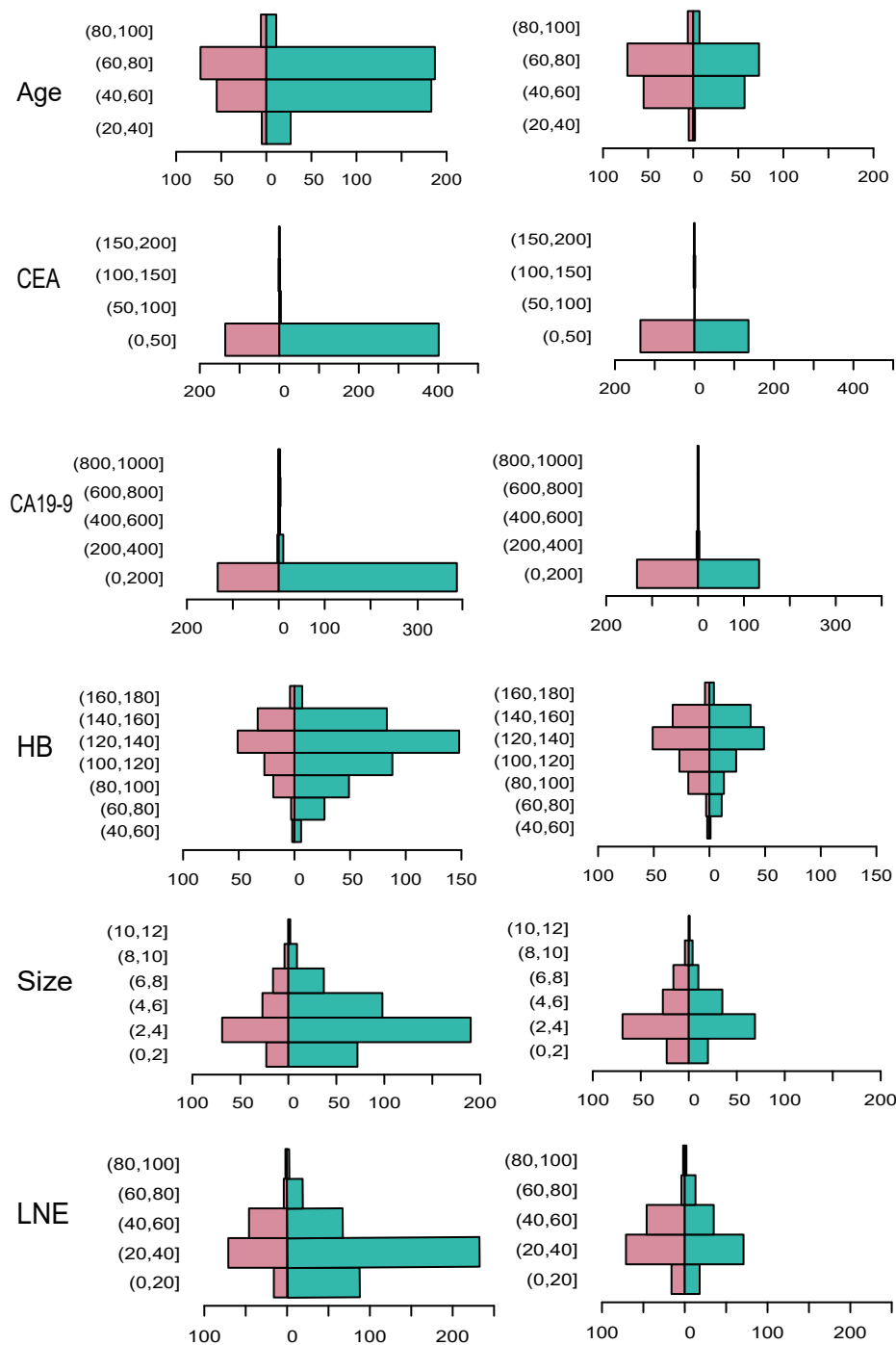

# Original

Grade

■ I/II ■ III

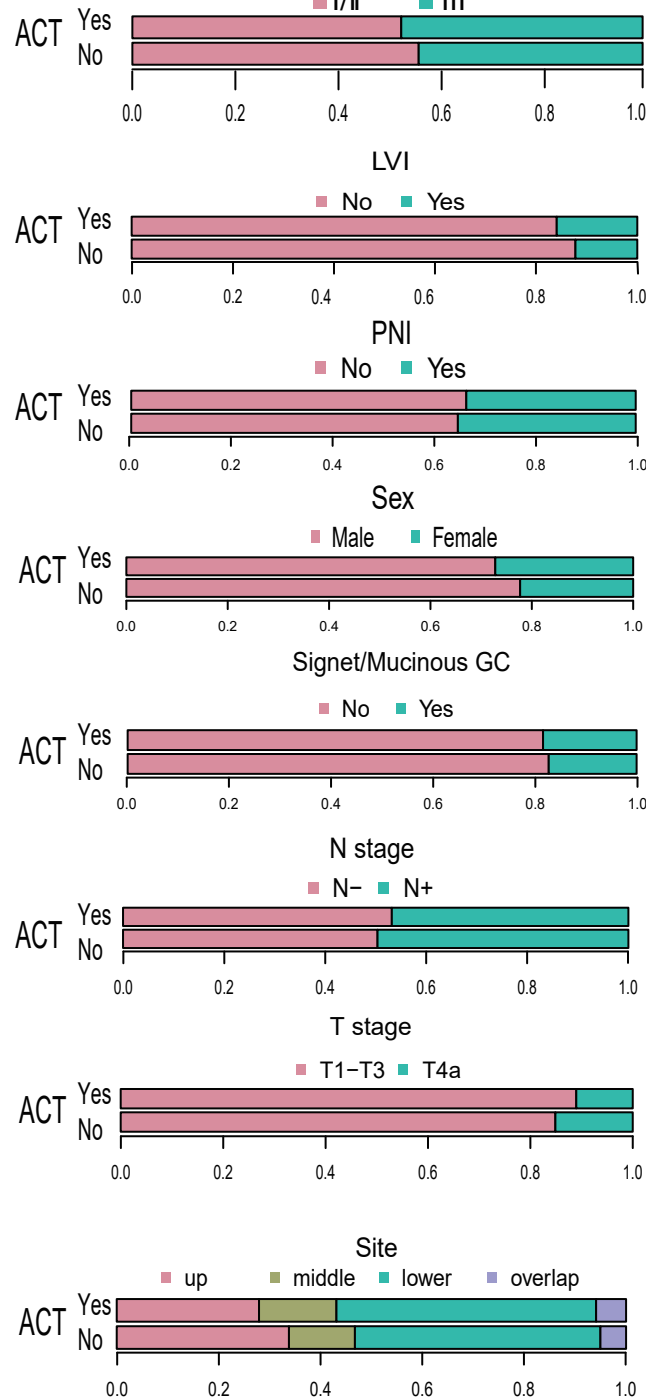

# Matched

Grade

■ I/II ■ III

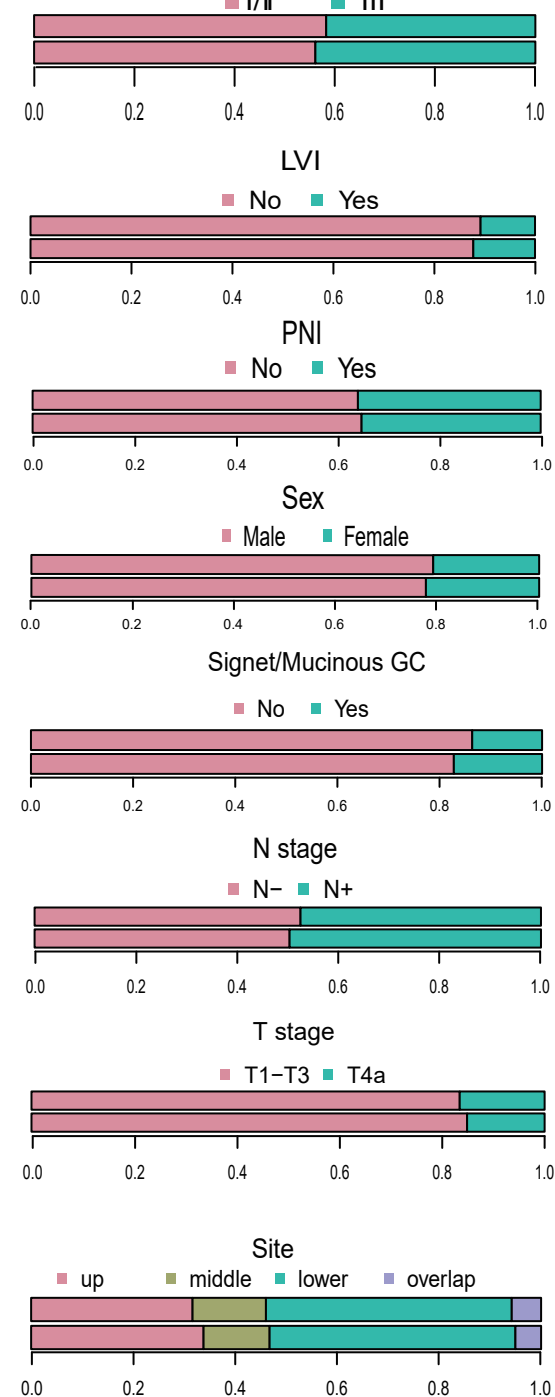

Supplement: Supplementary file 1 — Figure 1: Numerical variables and categorical variables before and after PSM. [file 12672_2023_663_MOESM1_ESM.pdf]

## Five-Year Survival ROC

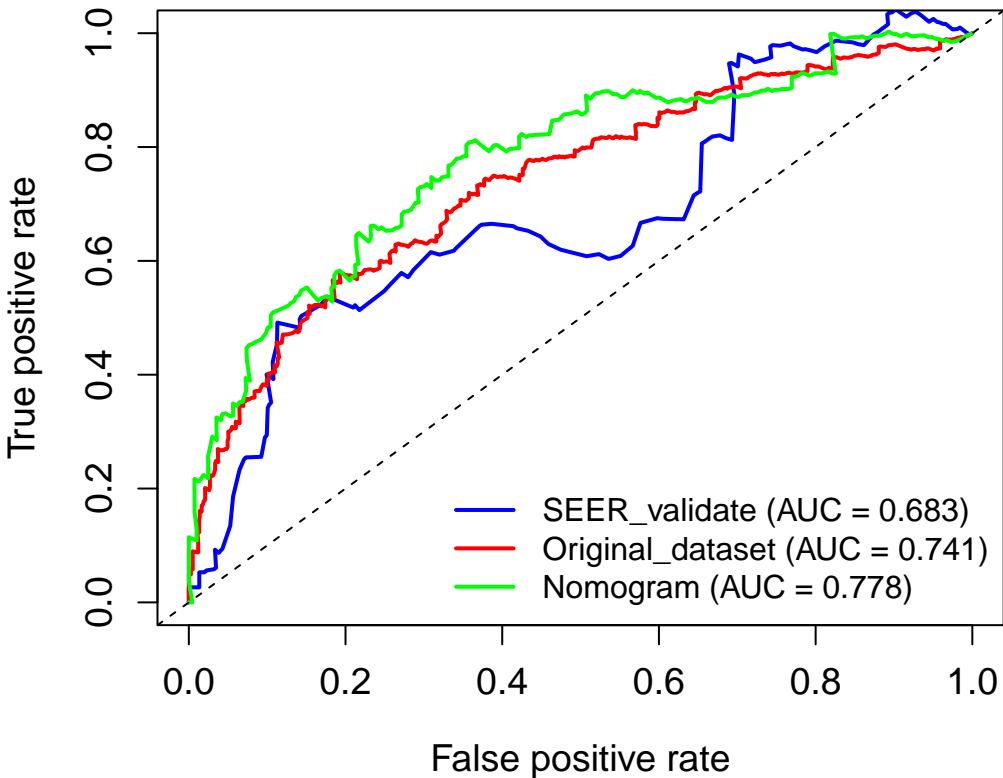

Supplement: Supplementary file 2 — Figure 2: ROC curves of train and validate datasets. [file 12672_2023_663_MOESM2_ESM.pdf]

## Five-year ROC (AUC = 0.778 )

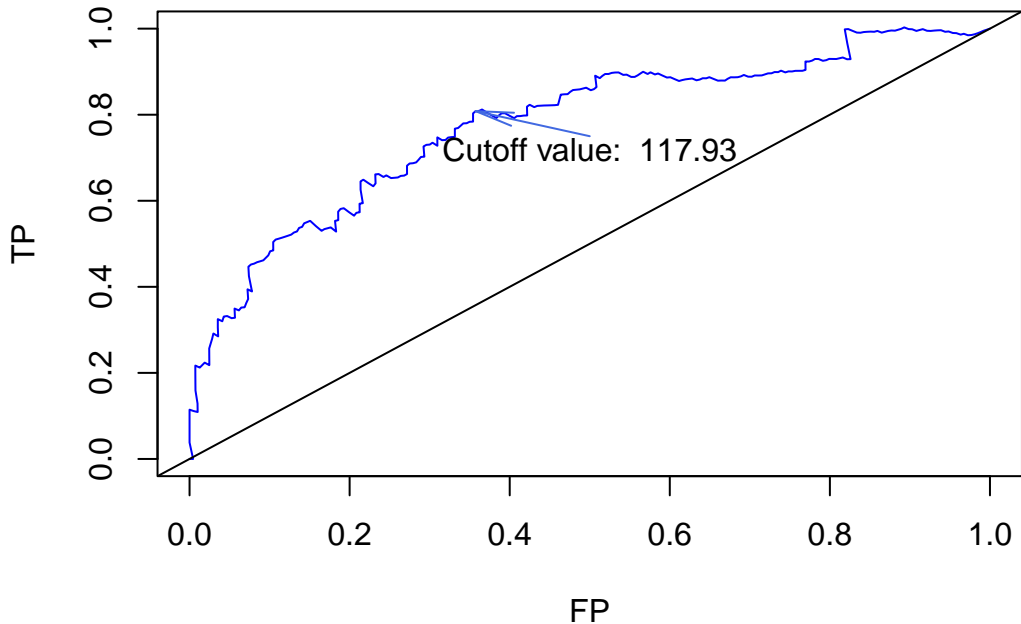

Supplement: Supplementary file 3 — Figure 3: Optimal cut-off value for risk stratification. [file 12672_2023_663_MOESM3_ESM.pdf]

# Hazard ratio

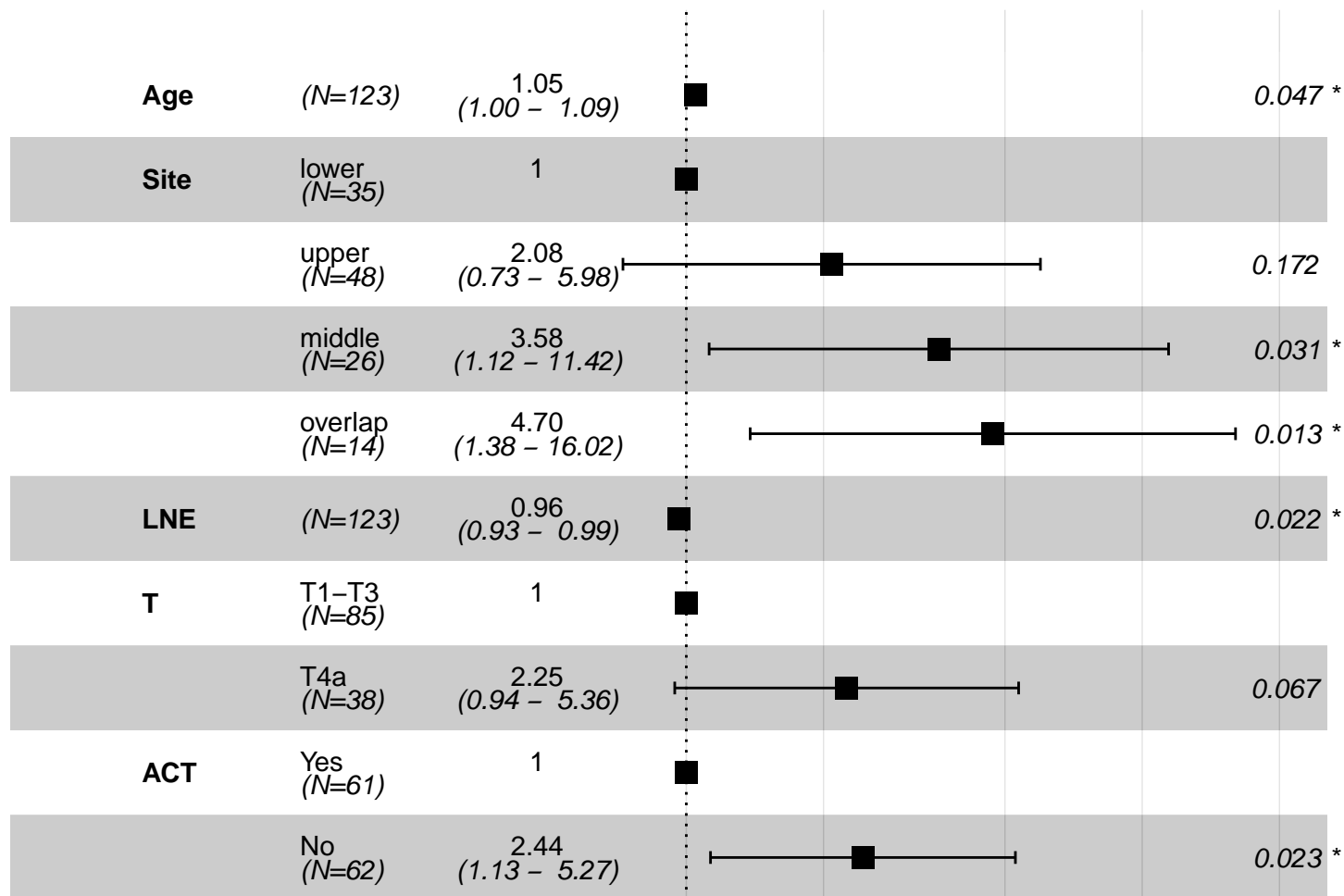

# Events: 32; Global p-value (Log-Rank): 0.0042117

AIC: 260.76; Concordance Index: 0.7

Supplement: Supplementary file 4 — Figure 4: Multivariate cox regression forest plot of the high-risk group. [file 12672_2023_663_MOESM4_ESM.pdf]

# Hazard ratio

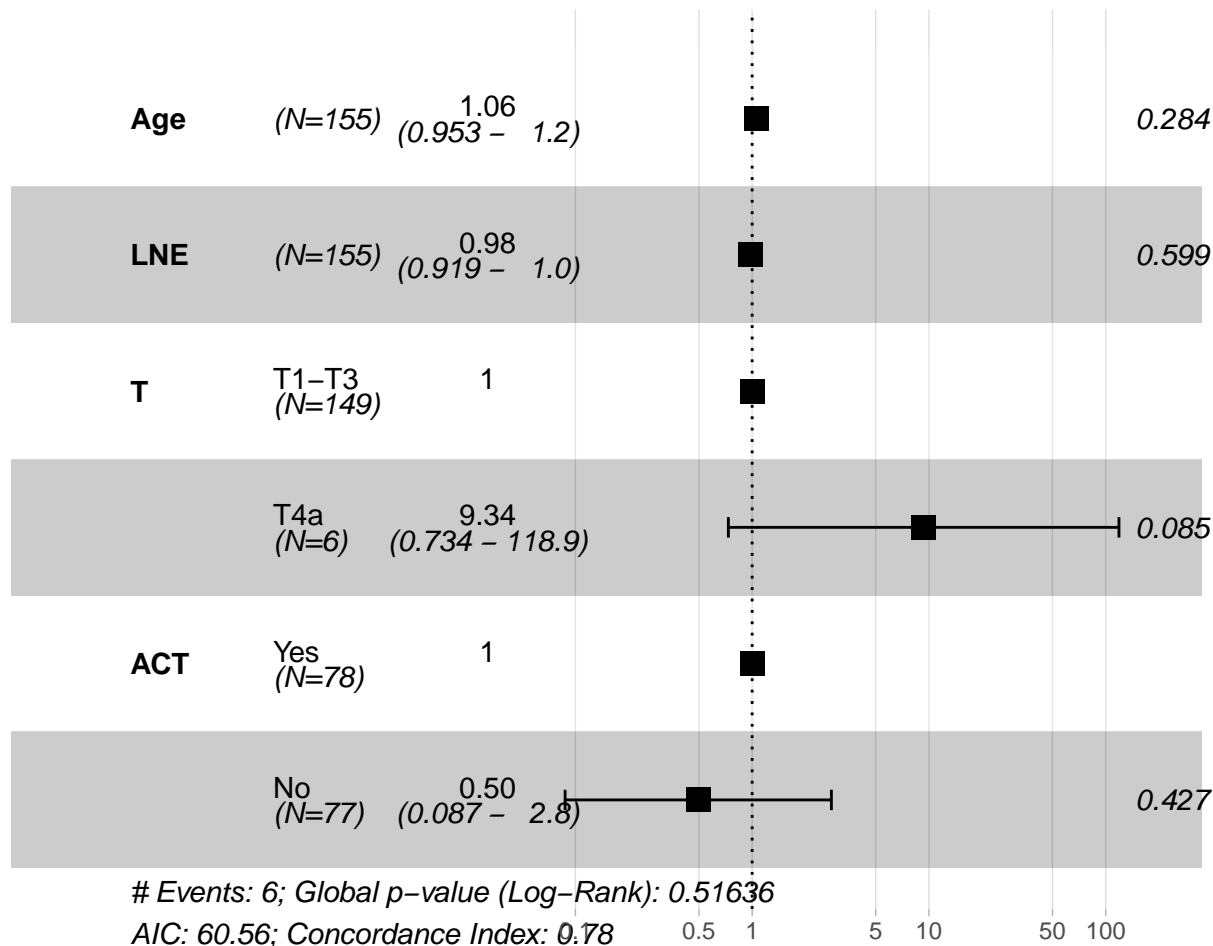

Supplement: Supplementary file 5 — Figure 5: Multivariate cox regression forest plot of the low-risk group. [file 12672_2023_663_MOESM5_ESM.pdf]
