# Supplementary material for: Prognostic factors and the necessity of chemotherapy for stage II gastric cancer: a model based on multicenter retrospective study
Source: Discov Oncol. 2023 May 8;14:58. doi: 10.1007/s12672-023-00663-w (PMC10167061; doi:10.1007/s12672-023-00663-w)
Supplement: Supplementary file 6 — Table 1: Characteristics before and after imputation. Table 2: Characteristics between high-risk and low-risk groups [file 12672_2023_663_MOESM6_ESM.docx]

Supplementary Table 1. Characteristics before and after imputation

| Characteristics | Original  Mean (SD) | Imputation  Mean (SD) | | T test  *P*-value | Wilcox test  *P*-value |
| --- | --- | --- | --- | --- | --- |
| **CEA (ng/ml)** | 5.52 (16.35) | | 5.38 (16.00) | 0.89 | 0.90 |
| **CA19-9 (U/ml)** | 41.95 (123.34) | | 40.51 (120.79) | 0.85 | 0.94 |
| **Hemoglobin (g/L)** | 122.16 (24.51) | | 122.35 (24.35) | 0.90 | 0.91 |
| **Tumor Size (cm)** | 4.08 (1.97) | | 4.08 (1.96) | 0.99 | 1.00 |
|  |  | |  |  |  |
|  |  | |  |  |  |
|  |  | |  |  |  |
|  |  | |  |  |  |
|  |  | |  |  |  |
|  |  | |  |  |  |
|  |  | |  |  |  |
|  |  | |  |  |  |
|  |  | |  |  |  |
|  |  | |  |  |  |
|  |  | |  |  |  |
|  |  | |  |  |  |

Supplementary Table 2. Characteristics between high-risk and low-risk groups

| Characteristics | High-risk group  Number (%) | Low-risk group  Number (%) | | *P*-value |
| --- | --- | --- | --- | --- |
| **Age (years)** | 69 (63–74) | | 58 (52.5 –63) | <0.001*** |
| **Sex** |  | |  | 0.37 |
| Male | 100 (81.3) | | 118 (76.1) |  |
| Female | 23 (18.7) | | 37 (23.9) |  |
| **CEA (ng/ml)** | 2.50 (2.00–4.08) | | 2.30 (1.29–3.59) | 0.01* |
| **CA19-9 (U/ml)** | 9.26 (6.11–22.50) | | 9.20 (4.55–17.90) | 0.37 |
| **Hemoglobin (g/L)** | 127 (107–138) | | 132 (111 – 144) | 0.13 |
| **Tumor stie** |  | |  | <0.001*** |
| up | 48 (39.0) | | 43 (27.7) |  |
| middle | 26 (21.1) | | 12 (7.7) |  |
| lower | 35 (28.5) | | 99 (63.9) |  |
| overlap | 14 (11.4) | | 1 (0.7) |  |
| **Tumor Size (cm)** | 4.00 (3.00–6.00) | | 4.00 (3.00–5.00) | 0.02* |
| **Grade** |  | |  | 0.13 |
| I/II | 77 (62.6) | | 82 (52.9) |  |
| III | 46 (37.4) | | 73 (47.1) |  |
| **Signet cell/Mucinous carcinoma** |  | |  | 0.40 |
| No | 107 (87.0) | | 128 (82.6) |  |
| Yes | 16 (13.0) | | 27 (17.4) |  |
| **Perineural invasion** |  | |  | 0.28 |
| No | 84 (68.3) | | 95 (61.3) |  |
| Yes | 39 (31.7) | | 60 (38.7) |  |
| **Lymph-vascular invasion** |  | |  | 0.53 |
| No | 111 (90.2) | | 135 (87.1) |  |
| Yes | 12 (9.8) | | 20 (12.9) |  |
| **T stage** |  | |  | <0.001*** |
| T1-3 | 85 (69.1) | | 149 (96.1) |  |
| T4a | 38 (30.9) | | 6 (3.9) |  |
| **N stage** |  | |  | 0.047* |
| N- | 72 (58.5) | | 71 (45.8) |  |
| N+ | 51 (41.5) | | 84 (54.2) |  |
| **Lymph nodes examined** | 27 (20–37) | | 41 (32–49.50) | <0.001*** |
| **Adjuvant Chemotherapy** |  | |  | 1.00 |
| Yes | 61 (49.6) | | 78 (50.3) |  |
| No | 62 (50.4) | | 77 (49.7) |  |
